# Adult vaccination as part of a healthy lifestyle: moving from medical intervention to health promotion

**DOI:** 10.1080/07853890.2019.1588470

**Published:** 2019-04-26

**Authors:** T. Mark Doherty, Giuseppe Del Giudice, Stefania Maggi

**Affiliations:** a GSK, Global Medical Affairs, Wavre, Belgium;; b GSK, Vaccine Research & Development Discovery Performance, Siena, Italy;; c CNR, Institute of Neuroscience - Aging Branch, Padua, Italy

**Keywords:** Ageing, frailty, immunosenescence, vaccination, healthy ageing, healthy lifestyle

## Abstract

As the global population ages, there is concern about the effect of an increased proportion of older individuals on the economic sustainability of healthcare systems and the social effects of an older society. Health authorities and advocacy groups in countries at the forefront of this trend are now developing strategies to ameliorate the social and financial effects of an ageing population. There is broad agreement that for both society and for the individuals, it is important to ensure that increasing lifespans are matched with increased “healthspans” – the number of years spent in good health. There is also growing consensus that vaccination is one of the tools that can play an important role in improving adult health – though currently vaccination coverage is often poor. This review focuses on two issues that consistently appear to be associated with under-vaccination: the low awareness of risk (and potential consequences) for vaccine-preventable diseases and a poor understanding of the value of improved vaccination coverage for adults. We suggest that understanding of vaccination as a health-promoting activity, rather than a medical intervention designed to prevent the spread of a specific pathogen – is a crucial step to improve vaccination uptake among adults (see Supplementary video abstract).

**Key messages**
- As populations age globally, we are seeing an increasing burden of vaccine-preventable disease in adults.
- Adult vaccination against some common diseases has been shown to dramatically improve health and quality of life for older people.
- Despite the attested benefits, vaccination coverage is almost always poor in adults, even in countries where access is free at point of care.
- In this article, we discuss what appears to a neglected issue in adult vaccination, that of personal autonomy. We argue that adult vaccination will only be successful if it respects individual autonomy and that this requires treating the choice to vaccinate as a public health issue akin to smoking cessation, exercise and healthy diet.

## Introduction

The global population is ageing rapidly, partly through persistent declines in birth rates and partly due to widespread improvements in life expectancy [1]. Vaccination has been one of the major drivers in this process, by reducing deaths from complications of co-existing chronic conditions and directly from infectious disease [2]. However, most vaccination programmes today are focused on reducing mortality and morbidity in children, even though the major burden of vaccine-preventable disease (VPD; Table 1) is in adults, not in children [6,7]. This focus appears to have been partly shaped by the demographics of the early-mid twentieth century, which were skewed towards younger, growing populations at the time when many national vaccination programmes were first being put in place. In addition, a focus on childhood vaccination was justified by high attack rates for many VPDs in infants and children. Finally, perceptions of cost-effectiveness may have played a role – it is often easier to reach infants and children through primary healthcare contacts, and they are a more homogenous group regarding response to vaccination. It can also be argued that saving the life of a young person who might live for many years may be a better use of resources than saving the life of an older person who might only live a few years more. This focus on childhood vaccination has left an impression in many minds that vaccination is an activity only relevant to children. This bias can be seen in the fact that adult vaccine coverage is almost always lower (and often much lower) than paediatric vaccine coverage in the same country, even for diseases where adult vaccination is recommended and fully reimbursed [8].

**Table 1.** Human vaccine preventable diseases [3–5]^a^.

Infection	EPI^b^ recommendation	Infection	EPI^b^ recommendation
Anthrax		Pertussis	✓
Cholera		Pneumococcal disease	✓
Dengue		Poliomyelitis	✓
Diphtheria	✓	Q fever	
Hepatitis A		Rabies	
Hepatitis B	✓	Rotavirus	✓
Hepatitis E		Rubella	
*Haemophilus influenzae* type b (Hib)	✓	Smallpox	✓
Human papillomavirus (HPV)		Tetanus	
Influenza		Tick-borne encephalitis	
Japanese encephalitis		Tuberculosis	✓
Malaria		Typhoid	
Measles	✓	Varicella (Chickenpox and Zoster)	
Meningococcal meningitis		Yellow Fever	
Mumps			

^a^ Vaccines listed are not all recommended or relevant in all countries. This list covers licensed vaccines which have been and widely used in humans, but does not include those which are no longer readily available such as the killed *Yersinia pestis* vaccine, or which have been used, but are not yet licensed, such as the Ebola vaccines.

^b^ The EPI is the Expanded Programme on Immunisation – a WHO programme established in 1974 to develop and expand immunisation programmes throughout the world. The vaccines listed are current as of 2018 (http://www.who.int/immunization/programmes_systems/supply_chain/benefits_of_immunization/en/).

Ultimately, low vaccine coverage in adults reflects the fact that adult vaccination has not been a public health priority for decades in the way that paediatric vaccination has been, and that the infrastructure for delivering vaccination – and vaccine education – for adults is not developed to the same extent. As noted by William Schaffner, President of the National Foundation for Infectious Diseases in the United States (US): “*Where we are with adult immunization is where we were 25 years ago with children immunization”* [9]. While this problem is not new, changing demographics are now raising the profile of VPDs in older patients alongside concerns about the impact of ageing populations on healthcare provision and healthcare budgets [10–12]. Vaccination is among the most effective of healthcare interventions, and improved vaccination coverage can potentially deliver significant decreases in healthcare utilization, and improved quality of life for older adults [12,13]. At the same time, more detailed tracking of burden of disease and more sophisticated health economic analyses suggest that the benefit of vaccination may in fact be generally under-estimated. The first part of this narrative review aims to introduce the effects that demographic changes are having on vaccination planning, discuss how vaccination has contributed in part to this demographic change, and introduce the increasing number of studies that suggests adult vaccination has broad health benefits beyond the prevention of a few targeted acute diseases. The second part of the review will introduce the concept of healthy aging, particularly with regard to vaccination, discuss why adult vaccination appears to consistently lag paediatric vaccination and suggest some potential routes to addressing this.

In particular, we want to emphasize two important concepts. First, there is no question that in many regions, barriers to adult vaccination exist in terms of access, affordability, reimbursement and infrastructure. These have been well discussed in the recent literature [14,15] and will not be the focus of this analysis. However, vaccine coverage remains poor in many countries where access to healthcare and vaccination are free at point of care, and vaccination is recommended: this implies that not all barriers to vaccination are not economic or medical, but instead may be psychological and/or educational [16,17]. We examine this hypothesis and suggest (based on responses of adults to surveys on vaccination) that an imperfect analysis of the personal risks and benefits of vaccination may decrease the likelihood of an adult to accept vaccination. Second, that addressing these psychological/educational barriers can provide synergistic improvements in quality of life and healthcare delivery that go beyond those normally associated with prevention of individual VPDs [18].

### Vaccine-preventable disease and the consequences of ageing

As we age, the progressive decline of immune responsiveness (immunosenescence) increases our risk for some infectious diseases. This heightened risk, together with other factor such as changes in our microbiomes (with possible dysbiosis – a profound disruption of the microflora – as a result of antibiotic treatment), the accumulation of co-morbidities (with their associated risks due to polypharmacy) and falling responsiveness to some antimicrobial therapies all increase the risk of serious illness, or even death from VPDs [19–22]. Social factors such as exposure through increased travel by older persons [23,24] or through clustering (for example, in long-term care facilities or community living) may further exacerbate risks. A recent analysis of the annual burden of VPD in the United States indicates the scale of the challenge [25]. There are more than 50,000 deaths attributed to VPD each year (99% of which are in adults [26]) and modelling suggested that VPDs cost the US approximately 9 billion USD in direct medical costs and lost productivity [25]. Roughly 79% of these costs were found to be due to disease in unvaccinated individuals [25]. Though no comparable analysis has been published yet, figures from the European Union suggest the burden of disease is probably of a similar magnitude [10,27]. These figures – though substantial – may be an under-estimate, since they are derived from cases directly attributable to VPDs and ignored the cost of much related illness. As discussed below, VPDs also contribute significantly to all-cause morbidity and mortality by increasing the risk for other conditions – particularly cardiovascular and cerebrovascular diseases – which are often not captured in the analysis of burden of disease.

The higher attack rates and/or increased severity of some VPDs in older adults means that without effective intervention, the costs associated with control of infectious disease are likely to escalate as the population ages and the most at-risk groups increase as a percentage of the population [18,20]. While the burden of disease directly attributable to VPDs in high and middle-income countries appears minor compared to that attributable to major non-communicable diseases, it appears to be rising. This increased burden of disease is accompanied by rising age at notification, consistent with the change being at least partly driven by the ageing of the population [18]. Vaccination for older adults is an obvious solution: it has been estimated that government investment in vaccination of adults over the age of 50 years, provides a more than four-fold return in economic benefits over the lifetime of the vaccinated cohort [28]. In addition, improved vaccination coverage can offer significant health benefits and quality of life improvements for older individuals. Despite this, countries generally are struggling to implement adult vaccination programmes: coverage remains well below recommended levels in almost every region and coverage rates are often either stagnant or even slightly decreasing [29,30]. And while the figures discussed here are mostly drawn from high-income countries, these issues are likely to be even more relevant for low-to-middle-income countries that are experiencing a rapid transition towards an older population, but which have even more constrained healthcare budgets and limited infrastructure for adult vaccination.

### Vaccination and the extension of life expectancy

The increasing proportion and the increasing absolute number of older people in the global population have been thoroughly covered in the recent literature and so needs little further discussion here [18,20]. It is, however, worth noting that the increase in life expectancy at the population level that we have observed has been driven by a reduction in premature mortality, rather than an extension of life *per se*. While average life expectancy has almost doubled, maximum human life expectancy (the age attained by the very oldest humans) has changed little, if at all, over the last century. One of the major drivers in this decline in premature mortality has been the elimination of infectious disease as a leading cause of death [31,32]. Of course, vaccination is not the only medical intervention to have contributed to increasing lifespans over the course of the last century. Public health interventions such as provision of clean water, better diet and improved living conditions have certainly played a major role, but declines in mortality directly associated with VPDs in vaccinated populations have been consistent and significant. It is estimated that reductions in infant mortality due to the elimination of previously common VPDs contributed substantially to the growth of Europe’s and North America’s populations in the nineteenth and twentieth centuries, with a detectable benefit in reduced mortality rates throughout life [31,32]. The declines in adult liver cancer attributable to hepatitis B, or in deaths from maternal tetanus, are but two examples [33,34]. In addition, significant reductions in adult mortality have been seen due to the effect of herd immunity and decreased transmission of diseases such as influenza and pneumococcus [35,36]. Indeed, it has been shown that the reduction of mortality attributed to pneumococcal vaccination of children in the US and the UK is primarily due to the reduction in transmission, leading to fewer deaths in older – unvaccinated – adults [35,36]. Similar beneficial effects on adult mortality have been documented by decreasing the transmission of influenza in school-age children [35,36]. However, inter-generational transmission of VPDs is not exclusively from children to adults. The group at highest risk of severe outcomes from pertussis is neonates and young infants, and in this case, transmission is usually from older family members. As expected, it appears that increased pertussis in adults (very likely due to waning immunity) correlates with an increased risk of infection, illness and death from pertussis in infants [36]. It is thus clear that the burden of VPDs needs to be seen in the context of a lifetime risk, and that can only be addressed by lifecourse vaccination.

### The full burden of vaccine-preventable disease

Overall, it has been estimated that effective childhood vaccination programmes have roughly halved crude mortality rates in children and infants [32] – an interesting observation, since this is a greater reduction than can be attributed to the elimination of direct mortality from VPDs themselves. Nonetheless, this observation has been remarkably consistent from country to country [32]. The root cause of this observation appears to be the physiologically damaging effect of some infections. Measles, for example, appears to increase all-cause mortality rates – particularly in children – by eliminating a significant proportion of pre-existing immunological memory, increasing susceptibility to other infections [37].

However, non-specific effects on morbidity and mortality from VPD are not limited to children. Influenza in adults significantly increases the risk of cardiac and cerebrovascular disease in the period immediately after the development of symptomatic disease and, while younger adults actually have the greatest relative increase in risks of stroke after influenza-like illness (due to low baseline levels of stroke in the uninfected population), the total burden of disease is highest in older adults [38,39]. Varicella-related disease also appears to increase the risk for cerebrovascular disease and myocardial infarct significantly and again, the burden of disease is greatest in older adults who develop zoster [40,41]. The precise mechanisms involved are only partially explained, though the finding of increased risk after infection is consistent across multiple populations. Some infections, such that caused by varicella zoster virus, may have a direct effect by infiltrating the walls of blood vessels and causing inflammation. It is also thought that systemic inflammation during influenza and influenza-like illness, may promote endothelial injury or a pro-thrombotic state in patients. These effects can be exacerbated by stressful events such as fever, exhaustion and dehydration [42]. Older, frail patients are at particular risk, given their lack of resilience, which may impede recovery after such infections. The same may be true of patients with co-morbidities such as chronic kidney disease or chronic obstructive pulmonary disease [42].

Vaccination can have a dramatic impact on these risks. Studies on the impact of influenza vaccination in older adults demonstrate reductions in the incidence of stroke and acute myocardial infarction of approximately 20% and studies in at-risk older patients with at least one co-morbidity show a clear dose–effect over multiple influenza seasons [43,44]. There are some indications that pneumococcal vaccination of older adults can also reduce the risk of cardiovascular disease, though the association is weaker than that reported for zoster or influenza [45,46]. Vaccination can markedly reduce demand for health services by older adults. In one prospective, randomized trial of adults 65 years of age or older, with at least one co-morbidity, vaccination against influenza and pneumococcus reduced intensive care and cardiac admissions in the vaccinated group by 41% and 55%, respectively, compared to the unvaccinated cohort, over the following 2 years. Overall mortality was also reduced by 35% [47]. The observed reduction in healthcare utilization and mortality was significantly larger than can be directly attributed to prevention to cases of influenza or Community-Acquired Pneumonia (CAP) alone. On analysis, much of the reduction in healthcare utilization was due to fewer admissions for cardiac and cerebrovascular disease, consistent with the effects of influenza discussed above. When the total burden of VPD – including downstream effects like cardiac disease and stroke – is considered, the case for improving vaccination coverage in adults appears to be compelling. However, the low level of vaccine coverage in most countries suggests that this message has not gotten through [8,48–50]. Multiple surveys have indicated that a frequent reason for adults to refuse vaccination against influenza is that the disease is not considered serious [17,51]. Since it is unlikely that many people regard admission to an intensive care ward or death as “non-serious,” the implication is that the potential severity of influenza – particularly in older adults – remains under-appreciated.

### Health, health promotion and healthy ageing

Health is generally considered to be more than just the absence of an identifiable disease. For example, a frail, elderly person who is unable to carry out the normal functions of daily living would not generally be considered as “in good health,” even in the absence of an acute illness. The WHO defines good health as “*a state of complete physical, mental and social well-being and not merely the absence of disease or infirmity*” [52] and healthy ageing as “*developing and maintaining the functional ability that enables well-being in older age*” [53]. Medical dictionaries typically use similar definitions, which suggests that even the broader definition of the morbidity due to VPDs does not capture their full effect, including decreased quality of life or on ageing-related declines in health. With regard to the latter, an increasing body of evidence suggests that exposure to some infectious diseases throughout life, even those which are resolved without obvious sequelae, may contribute to poorer outcomes in later life and potentially shorten life expectancy [54,55]. Certainly, many of the mechanisms involved in “inflamm-ageing” – the low-grade inflammatory immune response that appears to be a central feature of ageing – are those also intimately involved in protection from many infections [56]. As a result, it has been suggested that the overall burden of infectious disease throughout life may be one of the extrinsic factors that explain the variations in functional age seen between individuals [54]. A simplified version of the hypothesis is that ageing not only increases our susceptibility to physiological insults such as infectious disease (and reduces our ability to effectively respond to them), but that stressors such as infectious diseases promote biological reactions (i.e. inflammation), that may accelerate ageing. This hypothesis – while far from fully accepted – is consistent with findings from human studies such as the correlation of seropositivity to various pathogens with markers of elevated risk for cardiac disease, such as C-reactive protein, the association of chronic cytomegalovirus infection and reactivation with increased risk for frailty syndrome, or influenza-associated morbidity and mortality in older (65+) adults, the increased risk of atherosclerosis and stroke after some viral infections, or the increase in frailty seen after diseases such as acute lymphocytic leukaemia [57–60]. This hypothesis is also consistent with the observation that anti-inflammatory immunomodulators such as Rapamycin appear to extend life expectancy in mice, though the mechanism is not yet fully explained [61–63]. Existing evidence strongly suggests that VPDs contribute to a substantial burden of disease – beyond that directly attributable to illnesses such as acute influenza – and that vaccination throughout life can potentially reduce the risk of a wide range of ageing-related conditions. Better vaccination coverage might help to avoiding or at least deferring some of the negative results of ageing. This approach – the avoidance (as far as is practical) of the negative effects of ageing – is what is referred to as “healthy ageing” and it has the potential to be an effective counter – strategy to the risks posed to health systems by an ageing population.

### Why does adult vaccination lag paediatric vaccination?

Given the strong case to be made as to the benefits of vaccination in adults, it is perhaps puzzling that coverage levels are so poor. There remains little doubt that part of the reason is the lack of comprehensive support (including issues of cost and reimbursement) or infrastructure to deliver adult vaccination, but this does not appear to fully explain the gap. In addition to the structural barriers to adult vaccination such as availability, access and cost (summarized in recent reviews [14,15]), we would suggest that another rarely discussed problem is the perception in both the medical profession and the public at large that the death or ill health of older people attributable to infectious disease is to a certain extent “inevitable”. The death of an older person from pneumococcal disease, for example, may be considered unfortunate, while death from the same illness in a child is often described as a tragedy. An example of this thinking is the familiar name for pneumonia in older adults: “*the friend of the aged*” [64]. That name predates antibiotics, but even today, older adults hospitalized with CAP, still routinely die soon after discharge [65]. This is a matter of concern, given that overall rates of hospitalization for CAP are increasing as the population ages [66], even though many cases of CAP are potentially vaccine-preventable [67]. Despite the high burden of disease in older adults, it took an average of 55 months for European countries to introduce paediatric pneumococcal vaccination programmes for children after the initial market authorization of the vaccine – versus 158 months for adult vaccination programmes [8]. In addition, universal mass vaccination programmes for paediatric pneumococcal disease are both more common among European countries, and achieve much higher coverage than equivalent programmes for adults [8]. Even where adult vaccination programmes have had a head start in terms of initiation, they have tended to be rapidly outperformed by paediatric programmes in terms of coverage. In the US, for example, vaccination against influenza has been recommended for older adults for decades and has been reimbursed under Medicare since 1993 [68]. However, coverage among those over 65 was 65.3% during the 2016–2017 influenza season [48]. In contrast, recommendations for universal influenza vaccination covering children were only made in 2010 [69]. By the 2016–2017 season, vaccination coverage rates among children 6 months–4 years, at 70.0%, had already outstripped those for older adults, while rates among adults under 65 languished at 37.5%: just over half that of younger children [48]. In the UK, similar results have been obtained: 4 years after introduction of universal influenza vaccination for children, coverage rates among children aged 4–5 reached 62.6% in the 2017/2018 season, after increasing every year [49]. This already exceeds coverage among UK adults in risk groups (48.9%) and is rapidly approaching that in adults over 65 (72.6%) [50], even though adult influenza vaccination has long been a government priority in the UK. These findings suggest uptake of vaccines by paediatric programmes is more rapid and consistent than that of adult vaccination programs against the same disease, even in countries such as the UK and the US where adult vaccination has been well-resourced. In general, the data suggest that adult vaccination is not reaching its potential, even in countries where recommendations, infrastructure and funding are in place to broadly support adult vaccination. On a more global scale, the low coverage levels in adults are reflected in the low funding (and thus, presumably, priority) given to vaccination in general and adult vaccination in particular. In the western Europe, for example, spending on vaccination comprises less than 0.5% of healthcare budgets – often much less – and paediatric vaccination accounts for the lion’s share of this [70].

If vaccination of adults is to gain an increased share of healthcare resources, in an era where health budgets are broadly under pressure, it will need increased support. For this to happen, we argue that both the general population and healthcare professionals need to understand the benefits of vaccination, and accept the concept of vaccination as a preventive measure akin to exercise, healthy diet or medical risk reduction (e.g. the use of medications to reduce blood lipids). Currently, a substantial proportion of the resources lost from illness and treatment of VPDs in older adults could be prevented by vaccination, resulting in a considerable cost saving [25,28]. This argument becomes more compelling if we accept that instead of considering vaccination only in the context of preventing hospitalization and death, we should be looking at the benefit of vaccination to improve overall health and quality of life. These benefits can be expected to affect more than just the vaccinated person: improved quality of life in older adults will presumably also benefit their families and households, who often function as caregivers when older adults become ill or disabled. Extended healthy lifespans are also likely to provide broader economic benefits than just reduced medical costs, as healthy older people are more likely to remain engaged in the workplace and social activities [71]. However, achieving better vaccination coverage is almost certainly going to require changing the public perception of vaccination from a medical intervention (which we tend to think of as a response to illness) to a health-promoting activity (a preventive role). Ideally, vaccination needs to be seen by the public as something that lies within the scope of individuals to take charge of, for the benefit of their own health.

### What be done to boost coverage? Changing the public perception of vaccination

If we look at health in adults, and particularly older adults, there have been substantial improvements in the age-adjusted death rates of many non-communicable diseases, such as some cancers, stroke and heart attacks in high-income countries over the last few decades. The data suggest that these declines in mortality (and associated morbidity) have been driven partly by improved treatment options, but also partly by behavioural change [72–75]. This encompasses improvements in diet and exercise, reduction in smoking and improved participation in screening or treatment uptake. Adult vaccination programmes can perhaps learn from this: behavioural change can lead to substantial improvements in preventive health behaviours and ultimately in improved health outcomes. In some cases, such as the uptake of screening for some cancers, changes can be relatively rapid in at-risk populations and lead to significant improvements in outcome [76]. In these cases, and also in healthy aging, agency – the belief that the behavioural change contributes to the individual’s own health – is thought to be important [77]. This aspect: agency, or self-care, has also been found to be correlated with better health behaviours in older adults [78,79]. But experience from prior campaigns against poor health behaviours also sounds a warning note: fostering behavioural change is possible, but it can be difficult, and even where change can be affected, it may take significant time before improvements in health outcomes are discernible [80–83].

It is important to understand that when an adult refuses vaccination, this is not necessarily an irrational decision. Rather, the frequent references to risk and vaccine effectiveness in surveys of adults regarding vaccination suggest that it is often a cost/benefit analysis based on a mixture of psychological and educational factors – knowledge (or lack of knowledge) of disease risk versus the risk of side effects, a poor appreciation of consequences, lack of trust in authority figures, etc. [16,17,84]. However, the significant difference between the benefits attributable to vaccination demonstrated by clinical studies, as opposed to the low value attributed to vaccination by many members of the public, imply that in many cases, this analysis is flawed. If this is correct, then improving understanding – for example by a public health campaign to promote better understanding of the risks and benefits – has the potential to increase coverage.

When considering how to conduct public health campaigns to change established behaviours anti-smoking campaigns are an obvious place to start: they have perhaps been the most visible and among the most successful (in terms of changing health behaviours on a mass scale). They are certainly among the most-studied public health campaigns. However, they may be less relevant to campaigns to improve vaccination coverage since anti-smoking campaigns have been most successful in dissuading people from starting to smoke and often seek to stimulate negative responses towards the target activity [80]. Improving vaccination coverage requires encouraging an audience to take an action, or alter their existing habits and may, therefore, be more comparable to campaigns designed to promote positive attitudes towards the subject behaviour, such as exercise, or seeking screening for cancer (see Box 1). In addition, many of the most successful public health campaigns have incorporated legislative action such as bans on the sale of alcohol or cigarettes to minors, restriction on alcohol blood levels for drivers or restricting areas where smoking is permitted [85–88]. However, legislative action to reduce defined behaviours is intrinsically easier than legislative action to reduce non-compliance with a behaviour. As an example, while some countries have implemented mandatory vaccination policies (primarily for children [89,90]), these have often been controversial [91]. Mandatory vaccination for adults, with a few exceptions such as for military service, has typically been even more unpopular [92–95]. This means that campaigns to improve adult vaccination coverage – like campaigns to improve exercise or diet habits – have relied primarily on education. It is, therefore, worthwhile to look at what has – and has not – worked in this context.

**Box 1:** What makes a successful public health campaign [96–98]?

- An evidence base that provides a convincing case for taking action – for policy-makers, healthcare providers and for the general public.
- Political commitment, provision of resources and support for actions.
- An environment and infrastructure that supports the target audience to make the desired health behaviour changes.
- Acceptance and support of the message delivered by healthcare community.
- Delivery of a simple, effective message to a sufficient proportion of the audience to ensure the campaign’s messages and themes are broadly known and carry sufficient weight in the overall media landscape.
- Process analysis with rigorous monitoring, evaluation, to allow midcourse corrections and programme improvement.

There is good evidence that directly contacting or reminding individuals that they are eligible to receive a recommended vaccine can improve vaccine coverage rates, both in paediatric and adult settings [99,100]. However, such programmes are often resource-intensive, typically generate only incremental improvements and may only be effective in recipients who are already predisposed to vaccinate [100]. To reach the levels of coverage required to effectively interrupt transmission, it will almost certainly be necessary to also involve individuals who are hesitant about vaccination. Surveys indicate that the commonest reasons for hesitancy include factors such as an over-estimation of the frequency and severity of side‐effects associated with vaccination, a lack of confidence in effectiveness of the vaccine (particularly for influenza), an underestimation of the potential seriousness of the disease and a belief that the personal risk from disease is low [16,17,84]. In contrast, among the commonest reasons for accepting vaccination are a desire to be protected against disease and the belief that it is a social norm to vaccinate [95,101,102]. Looking at the factors associated with effective public health campaigns (Box 1), vaccination education appears to meet most or all of these criteria. There is ample data on the potential seriousness and the substantial risks associated with VPDs, as discussed above, and education on the potential consequences of VPDs may help to motivate some individuals to seek vaccination. However, education that focuses primarily on risk is not always effective and in some cases, it may even be counterproductive (see Box 2) [103,104]. A discussion of the risk of side effects – even if the conclusion is that these are minimal – may help to establish a mental landscape where vaccination is associated with risk [104] – particularly if it is conducted in a media environment where the focus of attention is on adverse events. Vaccine promotional programmes may, unwittingly, reinforce this negative message by just framing the benefit of vaccination in terms of avoidance of risk. A meta-analysis of interventions to improve vaccination uptake showed that the commonest approach was “*Information about Health Consequences*” where discussion of risk was a major theme [105]. Where a positive message is delivered, it often emphasizes the social benefit of vaccination (via herd or community protection). There is some indication that such altruistic appeals may improve vaccination rates [106,107]. However, in some cases at least, individuals may be more strongly motivated to vaccinate by the prospect of individual rather than societal benefit [108], and there is strong evidence that simply providing information about risks to health or the risk of failing to carry out the suggested health behaviour (i.e. vaccination) may not be sufficient to promote behavioural change [96,105]. Message framing is thus crucial: while risk – both of disease and of potential vaccine-related adverse events – needs to be addressed, it may be more productive to focus more on benefits (both to the individual and to society at large) and social norms.

**Box 2.** Media campaigns: when things go wrong

In any media campaign, it is essential that the intended message is delivered. There are multiple examples of media campaigns that have had unintended or even counter-productive consequences. This may be a particular risk when dealing with a topic that has a high social media profile such as vaccination, where the message delivered by health authorities will inevitably be seen in the context of ongoing media discussion [97]. In Vietnam, for example, hepatitis B vaccination coverage fell from 64.3% to 26.9% in the course of 2012–2013, despite a government campaign to promote vaccination. The apparent cause was media discussion on possible links between the vaccine and deaths. Even though investigation later showed no casual links, in the public mind the discussion increased fear of side effects. A subsequent study found that 68.2% of people asked became vaccine-hesitant after hearing about possible adverse events in the media, with 12.4% stating that they would refuse vaccination [109]. Similar findings have been reported from China, Italy and Canada [110–112].

Even where media debate is less fraught, campaigns can fail to have the desired effect – the anti-smoking “Think, don’t smoke” campaign may have increased intent to smoke among recipients, since while it discussed the health risks of smoking, it also portrayed smoking as an adult activity, an aspect potentially attractive to the mostly teenage audience [80]. A similarly negative outcome was reported by a randomized trial on vaccine messaging: showing images of sick children, and emphasising the severity of vaccine-preventable disease, increased anxiety regarding vaccination and fear of adverse events, apparently by establishing a mental association between vaccination and the disease it was intended to prevent. This appeared to actually decrease intent to vaccinate in some parents [104]. Additionally, although vaccine education appeared to increase understanding and decrease belief in a vaccine–autism link, it proved unpersuasive or even counter-productive to parents who already had formed a negative view of vaccination, emphasising the importance of context for assessing health information.

Interestingly, applying Behavioural Change Techniques (sometimes called “nudging”) to vaccine information may have a positive effect on individuals’ intent to vaccinate. Experimental studies of this approach suggest that presenting the outcome of vaccination as a benefit to be gained, rather than purely an avoidance of risk may improve intention to vaccinate [113]. Additionally, other studies assessing motivation for improved health behaviour (not just in vaccination) suggest that providing information on efficacy alongside avoidance of risk also improves intent to adopt the suggested health behaviour [114]. This is consistent with the theory of a rational approach to health behaviour (an individual will want to be certain that the proposed health behaviour is likely to be able to deliver the promised benefit before committing to it) since a commonly reported reason for not accepting vaccination is concern over efficacy. As an example, the strong demand experienced for the new recombinant zoster vaccine in the US [115] (even though an existing live-attenuated vaccine has been available for some years) may be attributable, in part to government recommendations and media coverage stressing the high level of efficacy of the newer vaccine [116]. Finally, studies of successful public health campaigns also indicate that to transform intent to action, there needs to be a supportive environment for the changes in health behaviour called for [97,117]. This can include things such as making access to information or to health services easier, a feeling that the desired activity is (or is becoming) a social norm and so on [80].

Based on these publications, we suggest that there is scope to improve intention to vaccinate among the general population, by public health campaigns that emphasize the positive effects of vaccination, alongside the more traditional discussion of disease avoided. Analysis of previous successful public health campaigns suggests that the most important element is simple, straightforward messages, framed to redefine the issue for the target audience. The concept of healthy ageing is ideally suited to this approach, since the core concept associated is a health gain (a positive benefit) and offers a straightforward path of action (seek healthcare advice on vaccination or request vaccination) for individuals to achieve the desired benefit [18].

## Proposals and conclusions

We, therefore, propose that the following points may be beneficial when considering programmes aimed at boosting adult vaccination:

- Present the full scope of risk that can be averted by vaccination: not just the risk of a specific disease like influenza, but also the associated risks. This should be presented as concrete potential outcomes, such as loss of income or employment, risk of hospitalization and risk of death, not just for individuals but for their families, friends, co-workers and society as a whole. This information needs to be personalized for the audience targeted: for example, adults in early employment or university have different risks and different concerns than retired persons. The risks attendant on vaccination should, of course always be covered, but they should not comprise a disproportionate amount of the message.
- Emphasize the benefits of choosing vaccination for the individual. As with discussions about avoidance of risk, the benefits presented should be tangible and relevant to individuals –improved quality of life, retention of the ability to live independently, financial or educational gains, improvement of life expectancy, etc. While the social benefits of vaccination are important, they should not be the sole focus and it may be beneficial to also emphasize the direct benefit to the vaccinated person. Given that multiple studies indicate that many people are loss-averse, presenting benefits as a way to prevent concrete losses may also be effective.
- Address concerns about efficacy. There is a substantial body of data indicating that vaccines are highly effective public health interventions. But it is particularly in this area that message framing is important and where the scope for improvement is possibly greatest. As an example, stating that influenza vaccination is only 30% effective at preventing disease is technically the same as stating that if you get vaccinated you reduce your risk of disease by about third. However, the former message is more likely to be perceived as highlighting a low level of effectiveness, while the latter highlights a personal health benefit.
- Provide plausible paths to action. If an individual is motivated to seek vaccination or advice on vaccination, it should – ideally – be as simple as possible for them to do so. Thus, public campaigns that aim to make vaccination a “health-promoting activity” will need to be matched by healthcare capacity to be effective – a message supported by the high levels of adult influenza vaccine coverage reached in parts of the UK, where educational campaigns have been matched by incentives and resources for vaccinators.
- Ensure there are mechanisms for feedback and assessment of impact. Not every programme aimed at enhancing vaccination coverage has been successful. Cultural and economic differences may mean that what is successful in one setting may not be feasible or effective in another. It is very clear from a review of the literature that what works – and why – is not always apparent, even though substantial resources have been committed. To ensure that these resources are used wisely, it is essential to define and monitor the desired outcomes.

As we have attempted to show in this review, the benefits of adult vaccination are substantial, and the need for effective vaccine programmes in adults will only grow in the near future [12,18]. However, the degree to which adult vaccination programmes lag paediatric programmes in terms of coverage – even though the burden of VPDs lies primarily in adult population – suggests that a paradigm shift is required [26,118]. Based on the evidence available, promoting life course vaccination and healthy ageing concepts, where vaccinations are considered as part of a package of health behaviours designed to promote health and well-being in adults and older adults, rather than being seen primarily as measures to prevent transmission of disease from specific pathogens, may be a way to generate such a paradigm shift. This approach will require an understanding and acceptance of this viewpoint by the general population and health authorities alike. At the same time, it must be admitted that the quality of research in this area is generally incomplete, and that a better understanding of what motivates people (adults in particular) to choose to vaccinate is required. How well healthcare systems manage to improve adult vaccine coverage is likely to have major implications on how well they perform in the coming decades, and also on the health of their ageing populations.

## Supplementary Material

Supplemental Material
